# A strong early acting wound‐inducible promoter, RbPCD1pro, activates cryIAc expression within minutes of wounding to impart efficient protection against insects

**DOI:** 10.1111/pbi.13071

**Published:** 2019-02-26

**Authors:** Saurabh Prakash Pandey, Amar Pal Singh, Shruti Srivastava, Krishnappa Chandrashekar, Aniruddha P. Sane

**Affiliations:** ^1^ Plant Gene Expression Lab CSIR‐National Botanical Research Institute Lucknow India; ^2^ Academy of Scientific and Innovative Research (AcSIR) Ghaziabad India; ^3^ Genomics and Molecular Biology Division CSIR‐National Botanical Research Institute Lucknow India; ^4^ Present address: National Institute for Plant Genome Research New Delhi 110067 India; ^5^ Present address: IARI Regional Centre Aundh, Pune 411067 India

**Keywords:** insect resistance, constitutive promoter, GUS, jasmonic acid, *Bacillus thuringiensis*, tomato

## Abstract

The expression of insecticidal proteins under constitutive promoters in transgenic plants is fraught with problems like developmental abnormalities, yield drag, expression in unwanted tissues, and seasonal changes in expression. *RbPCD1pro,* a rapid, early acting wound‐inducible promoter from rose that is activated within 5 min of wounding, was isolated and characterized. Wounding increased transcript levels up to 150 and 500 folds within 5 and 20 min coupled with high translation as seen by histochemical GUS enzyme activity within 5–20 min. *RbPCD1pro* was activated by both sucking and chewing insects and showed wound‐inducible expression in various aerial tissues of plants representing commercially important dicot and monocot families. The promoter showed no expression in any vegetative tissue except upon wounding. Functionality of *RbPCD1pro* was tested by its ability to drive expression of the insecticidal protein gene *cryIAc* in transgenic *Arabidopsis* and tomato. Strong wound‐inducible CryIAc expression was observed in both plants that increased 100–350 fold (*Arabidopsis*) and 280–600 fold (tomato) over the unwounded background within 5 min and over 1000–1600 fold within 20 min. The unwounded background level was just 3–6% of the *CaMV35S* promoter while wound‐induced expression was 5–27 folds higher than the best *CaMV35S* line in just 5 min and 80‐fold higher in 20 min. Transgenic plants showed strong resistance even to larger fourth instar larvae of *H. armigera* and no abnormalities in development and general plant growth. This is one of the earliest acting promoters with wide biotechnological application across monocot and dicot plants.

## Introduction

The large scale destruction of crop plants by various chewing and sucking insects has necessitated the development of chemical as well as biotechnological means to prevent damage. One of the most prevalent environment‐friendly biotechnological approaches for protection against Lepidopteran insects has been the development of transgenic plants expressing the *cryIAc* gene and its variants from *Bacillus thuringiensis*. These insecticidal protein genes have most commonly been expressed under strong ubiquitously expressing promoters like CaMV35S, ubiquitin, actin, rbcS etc. and prevent insect attack by maintaining continuous high level expression of the toxin protein (Cao *et al*., 2002; Nayak *et al*., 1997; Tang *et al*., 2006; Tu *et al*., 2000; Ye *et al*., 2001; Ye *et al*., 2009; Zhao *et al*., 2014).

Despite their success, constitutive promoters are not the best choice for driving insecticidal toxin gene expression for several reasons: (i) they ensure continuous expression of the toxic protein in most tissues, even in the absence of the insect, thus entailing a huge metabolic cost on the plant that may affect yield (Breitler *et al*., 2004; Gurr and Rushton, 2005; Kim *et al*., 2008; Xia *et al*., 2010). (ii) expression of the toxic protein affects plant development with many transgenic plants showing abnormalities in certain developmental aspects or failing to survive the initial stages of tissue culture (Bano‐Maqbool *et al*., 1998; Barton *et al*., 1987; Breitler *et al*., 2004; Diehn *et al*., 1996; Koul *et al*., 2014; Kranthi *et al*., 2005; Rawat *et al*., 2011; Rocher *et al*., 1998; Sachs *et al*., 1998). (iii) many constitutive promoters like the *CaMV35S* or the maize ubiquitin promoter are not truly constitutive since they show developmental and seasonal changes and expression may taper down towards flowering or in certain tissues making these susceptible to attack (Kranthi *et al*., 2005; Wu *et al*., 2002). (iv) continuous expression of the protein increases fears of the possibility of rapid development of resistance due to selection pressure on the insects (v) Expression of insecticidal proteins in seeds and embryos of rice or other edible plants (Breitler *et al*., 2001; Wu *et al*., 2002) may act as a psychological deterrent for consumers.

Inducible promoters, especially wound‐inducible promoters, provide an attractive alternative to constitutive promoters because of their ability to reduce unnecessary background expression of the toxic protein and prevent possible abnormalities from their expression. Much research has led to isolation of several wound‐inducible promoters from different plants. These include the *AoPR1* promoter from *PR1* gene of *Asparagus officinalis* (Warner *et al*., 1993), the *mpiC1* promoter from a maize protease inhibitor gene (Cordero *et al*., 1994), the *fib* gene promoter from bell paper (Chen *et al*., 1998), the *win3.12* gene promoter from Populus (Hollick and Gordon, 1995; Yevtushenko *et al*., 2004), the *Shpx6b* peroxidase promoter from the forage legume, *Stylosanthes humilis*, (Perera and Jones, 2004), the *OsDof1* promoter of rice (Park *et al*., 2014) and others. A few of these such as *AoPR1* and *mpiC1* have been used for expression of CryIAc‐type proteins or protease inhibitors with varying success (Girijashankar *et al*., 2005; Gulbitti‐Onarici *et al*., 2009). A drawback with some of these has been the relative delay in their induction (from a few hours to even days for maximum activation), the reduced level of transgene induction or restricted specificity in heterologous systems (Wilmink *et al*., 1995; Zhang *et al*., 2013) thereby limiting their application for general use.

An early acting promoter, activated transcriptionally and translationally within minutes of wounding, has the potential to stall insect attack beyond a few nibbles and should ideally be effective not only against newly hatched larvae but also against larger larve of the 3rd and 4th instar stages and should stay silent in the absence of insect attack. Here, a strong wound‐inducible promoter, activated early within 5–20 min of mechanical and insect wounding is described. It is responsive to both chewing and sucking insects and functions in different tissues in several commercially important families. Functional validation through expression of the CryIAc protein in *Arabidopsis* and tomato shows its potential for use in several plants against insects.

## Results

### Characterization of the wound‐inducible *RbPCD1* promoter

The wound‐inducible nature of *RbPCD1* promoter was observed during study of abscission‐related *cis* elements in the promoter of a petal abscission up‐regulated gene of rose (Amar Pal Singh, 2011). A 523 nt region, upstream of the initiation codon, governing wound‐inducible *GUS* expression (Patent No 3866/DEL/2014 filed and WO2016103279A1) was explored in further detail in *Arabidopsis* and other plants.

**Figure 1:**
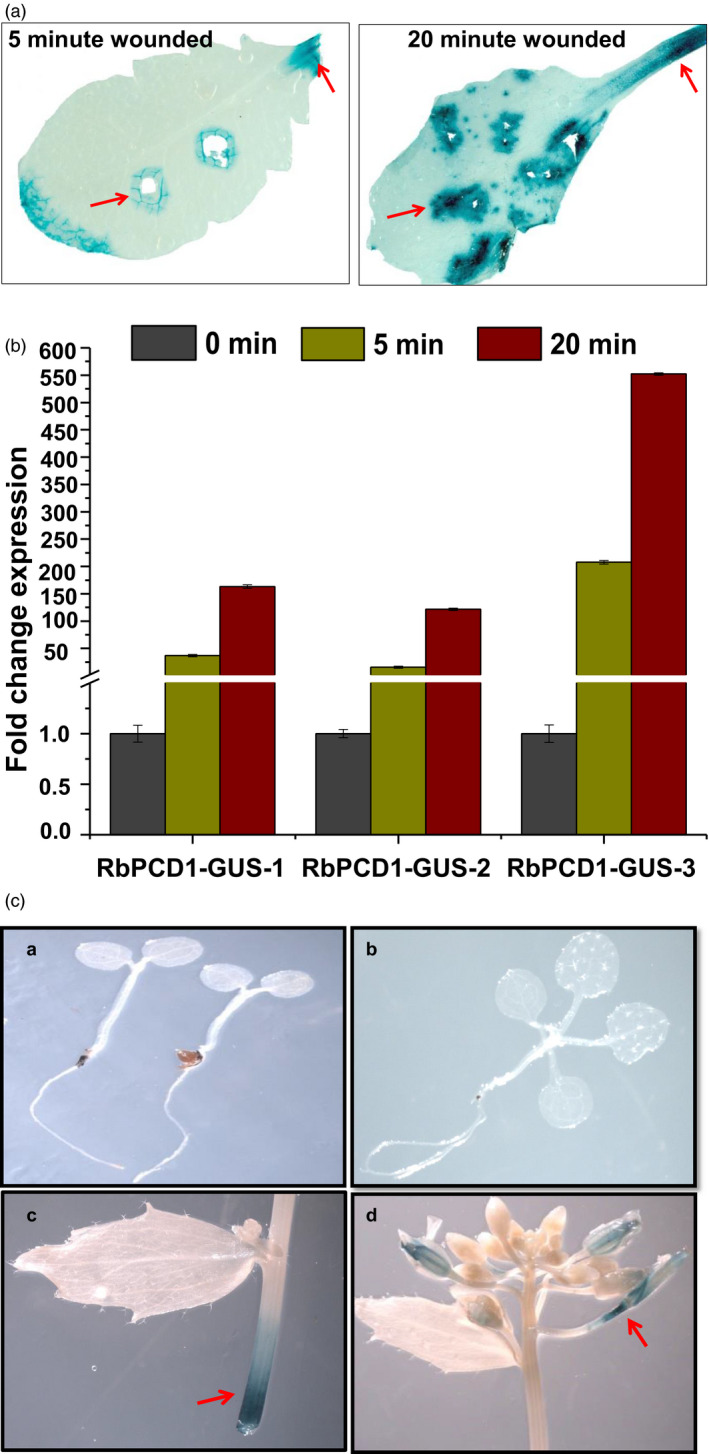
Wound‐inducible expression of *GUS* under the *RbPCD1* promoter in transgenic *Arabidopsis* plants. (a) Histochemical GUS staining of transgenic *Arabidopsis* leaves expressing *RbPCD1pro*:: *GUS* at 5 and 20 min after mechanical wounding. Arrows indicate the site of mechanical wounding or damage. GUS analysis was carried out in presence of cycloheximide. (b) qRT‐PCR analysis of relative transcript levels of *GUS* after mechanical wounding in transgenic *Arabidopsis* expressing *RbPCD1pro*:: *GUS* **.** Analysis was carried out in three independent transgenic lines. *AtUBIQUITIN10* was used as internal control. (c) Histochemical analysis of *GUS* activity in transgenic *Arabidopsis* expressing *RbPCD1‐pro::GUS* in various stages of plant development. (a) Two leaf stage (b) Four leaf stage (c) Excised stem (d) Flowering stage.

A time‐course analysis of wound induction of the *RbPCD1* promoter was carried out using leaves of more than five independent homozygous transgenic *Arabidopsis* lines expressing *RbPCD1pro::GUS*. Intact leaves were wounded by rapid punctures and kept for 5 and 20 min. This was followed by color development in the presence of cycloheximide to ensure that the observed color only represented the protein synthesized during the 5–20 min time course of wounding and not later during color development. Histochemical *GUS* assay of wounded leaves showed an intense blue color around the damaged area as well as the petiole tip from where leaves were detached (Figure 1a). No color was seen in the unwounded regions.

For further validation of the wound responsiveness of *RbPCD1pro*, a time course analysis of transcript accumulation of *GUS* mRNA in response to mechanical wounding was performed at 5/20 min after wounding. A rapid increase in *GUS* transcript levels, ranging from 50 to 150 folds within 5 min and from 150 to 550 folds within 20 min of wounding (compared to control), was observed in all lines suggesting that the promoter responded strongly to mechanical wounding (Figure 1b).

In order to examine the spatiotemporal expression patterns of *RbPCD1pro*::*GUS*, whole transgenic plants were histochemically stained at different developmental stages from seedling to flowering. No detectable *GUS* expression was seen in any plant part in the absence of wounding regardless of the stage of development except in the inflorescence and abscission zones (Figure 1c). The promoter was activated upon wounding at all stages (except senescent leaves) and in all tissues except root where no *GUS* expression could be observed even upon wounding (data not shown).

### *RbPCD1pro* is induced by both chewing and sucking insects and activated in dicots and monocots

**Figure 2:**
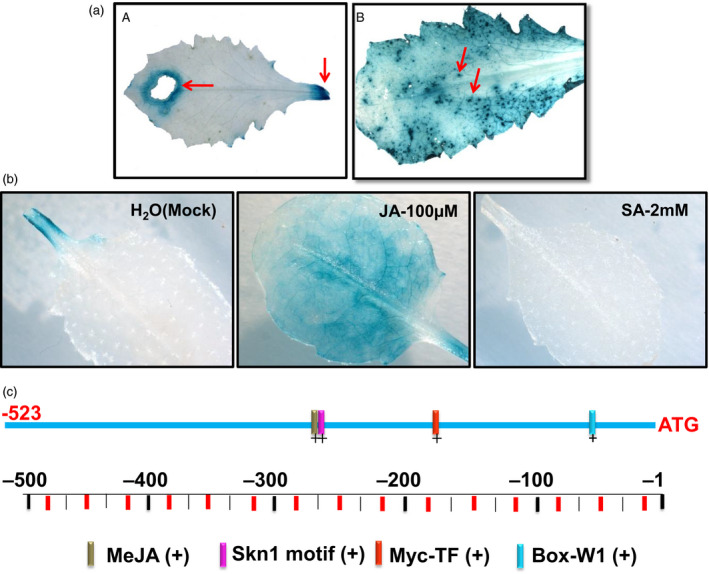
*In silico* analysis of the *RbPCD1* promoter and its response to insect wounding and different hormones. (a) Activation of *RbPCD1pro::GUS* by insect wounding. Histochemical GUS staining of transgenic *Arabidopsis* leaves was carried out after exposure to the chewing insect, *Helicoverpa armigera* (A)*,* and the sucking pest *Myzus persicae* (B). The arrow in the left panel indicates the site of insect bite while the blue dots in the lower panels indicate sites of feeding by the aphids. (b) Expression of *RbPCD1pro::GUS* in response to Jasmonic acid and salicylic acid in transgenic *Arabidopsis*. Leaves were sprayed with JA (100 μm)/SA (2 mm)/water, kept for 3 h and GUS expression checked. (c) *In silico* analysis of putative *cis* elements in the 523 nt region of the *RbPCD1* promoter by PLACE.

The *RbPCD1* promoter was next tested for its ability to respond to insect wounds. Leaves of independent transgenic *Arabidopsis* plants harbouring *RbPCD1pro*::*GUS* were fed upon by *Helicoverpa armigera,* a chewing insect, or exposed to the aphid, *Myzus persicae,* a sucking pest. A strong blue color was observed around the area damaged by both insects (Figure 2a) showing that the promoter responded to wounding by both chewing and sucking pests.

The promoter was next tested for wound response in different plants of economic importance that included dicots like chickpea, cotton, tobacco, rose, and a monocot Gladiolus by transgenic means (chickpea) or by agro‐inoculation (all other plants). Strong wound‐inducible *GUS* expression, only at the site of wounding, was seen in transgenic chickpea leaves as well as agroinjected cotton sepals, rose petals, *Gladiolus* tepals, and tobacco leaves (Figure S1) indicating that the promoter could respond to wound signals in a variety of plants belonging to different families.

### The *RbPCD1* promoter is responsive to JA but suppressed by SA

Wound responses are known to be regulated by both JA and SA, with JA activating wound responses and SA suppressing JA effects (Koornneef *et al*., 2008). To check this, a study of *RbPCD1pro* induction by JA and SA (in the absence of wounding) was performed. As shown (Figure 2b), JA treatment could induce *RbPCD1pro,* albeit weakly, compared to that observed by wounding. Interestingly, SA seemed to suppress the promoter since no GUS expression could be seen even at the point of petiole excision.

An *in silico* analysis of the 523 nt region of *RbPCD1* promoter was performed using PLACE (http://www.dna.affrc.go.jp/PLACE/signalscan.html). The analysis revealed several putative *cis*‐acting regulatory elements such as a Myc‐TF binding site, a JA responsive element, W‐boxes for WRKY transcription factor binding and others (Figure 2c).

### *RbPCD1pro* drives *cryIAc* expression in a strong wound‐inducible manner and protects transgenic *Arabidopsis* plants from insect larvae

In order to test the comparative efficacy and functionality of the *RbPCD1* promoter, constructs expressing *cryIAc* under the *RbPCD1* and *CaMV35S* promoters were introduced into *Arabidopsis* (both promoters) and tomato (only *RbPCD1pro*) as described in methods. Three independent T_3_ generation lines of transgenic *Arabidopsis*, homozygous for the transgene (AtPCDCry 3‐3‐1, AtPCDCry 4‐2‐1, and AtPCDCry 6‐1‐2), and tomato (SlPCDCry 3, SlPCDCry 9, and SlPCDCry10, T_1_ generation) were studied for wound‐inducible protein production and insect bioassay and also monitored for possible abnormalities in leaf shape, size, and plant growth.

**Figure 3:**
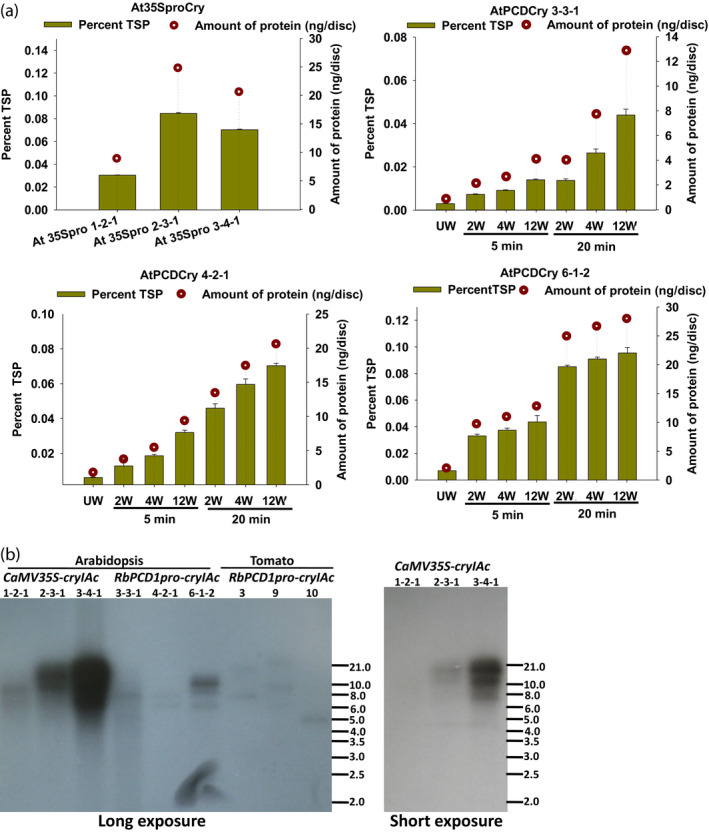
Copy number and expression of *cryIAc* under the *CaMV35S* and the *RbPCD1* promoters in different transgenic *Arabidopsis* lines. (a) Estimation of CryIAc in different transgenic lines under the *CaMV35S* and *RbPCD1* promoters. For *CaMV35S* lines, expression was estimated in leaf discs (177 mm^2^) by DAS‐ELISA using CryIAc specific antibodies. For wound‐inducible expression in *RbPCD1pro::cryIAc* plants, leaves were wounded with various punctures (2, 4, and 12), kept for 5 or 20 min on the plant and leaf discs excised from the plant. Green bars represent %TSP on left *Y*‐axis and circles represent amounts in ng/disc (177 mm^2^ surface area) on right *Y*‐axis. UW‐unwounded, W‐ wounded. (b) Southern blot analysis for copy number estimation of *cryIAc* in transgenic *Arabidopsis* and tomato lines. DNA from all independent transgenic plants expressing *cryIAc* under the *CaMV35S* (*Arabidopsis*) and *RbPCD1* (*Arabidopsis* and tomato) promoters was digested with HindIII. Blotting and hybridization was carried out as described in methods using ^32^PdATP‐labeled *cryIAc* (that lacks an internal HindIII site). Each band represents an independent insertion event.

For transgenic *Arabidopsis*, a quantitative estimate of the CryIAc protein produced in response to varying puncture wounds (2, 4, and 12 wounds) was first obtained in leaves on transgenic plants after 5 and 20 min. As shown (Figure 3A), basal level expression of CryIAc in *CaMV35Spro* plants ranged from 8.9 to 24.8 ng/disc corresponding to 0.05 (lowest) to 0.14 ng/mm^2^ (highest expressing plant). The basal level in transgenic *RbPCD1pro*::*cryIAc* lines was 0.005, 0.01, and 0.011 ng/mm^2^ in AtPCDCry lines 3‐3‐1, 4‐2‐1, and 6‐1‐2, respectively (Figure 3A). This was just about 3%–6% of the best *CaMV35S* line (0.14 ng/mm^2^). However, within 5 min of wounding, CryIAc expression increased to 0.628, 0.971, and 3.83 ng/mm^2^ in AtPCDCry lines 3‐3‐1, 4‐2‐1, and 6‐1‐2, respectively. This represented an increase in 4.5–27 folds over the highest expressing *CaMV35S* line and about 100–350 folds over the unwounded background. By 20 min, the expression increased further by 11–82 folds (1.572, 5.83, and 11.442 ng/mm^2^ in AtPCDCry lines 3‐3‐1, 4‐2‐1, and 6‐1‐2, respectively), over the *CaMV35S* line and 315–1040 folds over the unwounded background. An increase in the number of wounds from 2 to 4 or 12 led to a higher accumulation of protein although the increase was not linear (Figure 3a). A longer period of wounding from 5 to 20 min led to a much greater increase in CryIAc accumulation, the difference being about threefold between 5 and 20 min. Thus, the *RbPCD1* promoter could maintain the toxic CryIAc protein at a much lower basal level than the *CaMV35S* promoter but at a much higher induced level (up to 80 folds higher) compared to the best *CaMV35S* line and 100–1000 folds higher than the unwounded background level within 5–20 min. Since experiments were carried out in T_3_ generation plants, it also indicated that the wound‐inducible expression was maintained at least up to the third generation.

To ensure that the differences in expression between *CaMV35Spro* and *RbPCD1pro* plants were not due to copy number differences, a Southern blot analysis of the lines was carried out. As shown (Figure 3B), the number of *cryIAc* copies ranged from 1 to 3 in the *CaMV35S* lines (1 in 1‐2‐1, 2 in 2‐3‐1 and 3 in 3‐4‐1) and from 1 to 4 (1 in 3‐3‐1, 2 in 4‐2‐1 and 4 in 6‐1‐2) in the transgenic *Arabidopsis RbPCD1pro* lines indicating that copy number was not responsible for the differences in expression between *CaMV35pro* and *RbPCD1pro* lines.

To ascertain the efficacy of the CryIAc protein produced under the wound‐inducible *RbPCD1pro*, an insect bioassay was performed on transgenic *RbPCD1pro::cryIAc* plants. Both, detached leaf and whole plant insect bioassays, were conducted on *Helicoverpa armigera* larvae at four developmental stages (1st–4th instar) in independent sets of experiments.

**Figure 4:**
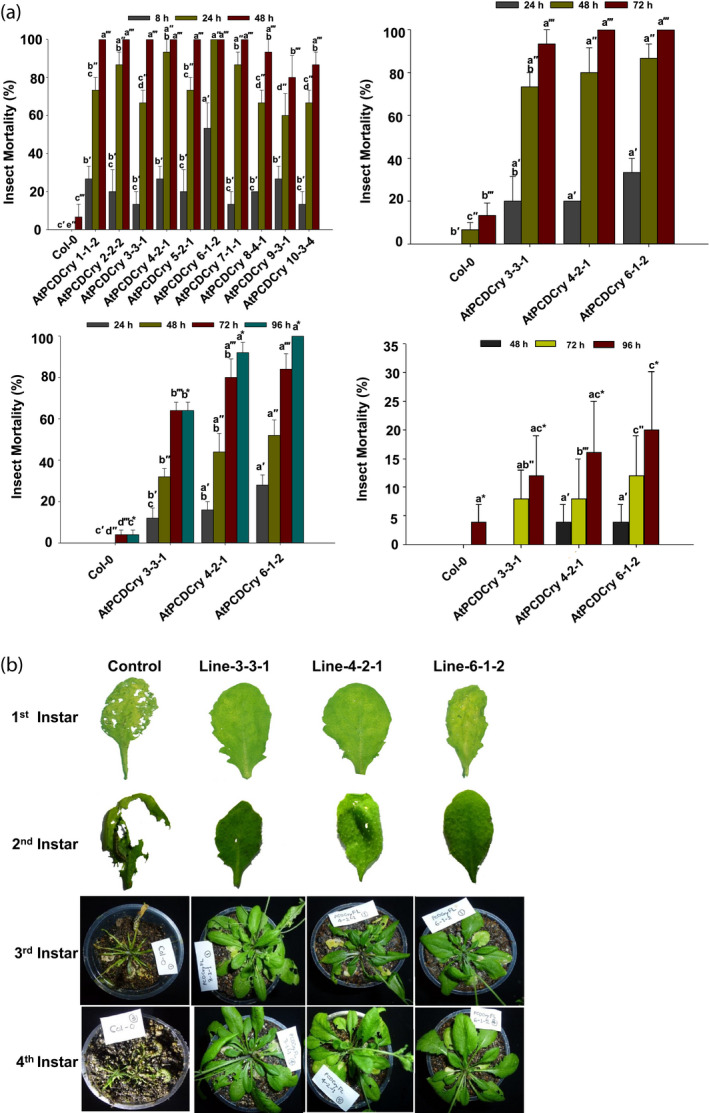
Efficacy of transgenic *Arabidopsis* plants expressing *cryIAc* under the *RbPCD1* promoter against *H. armigera* larvae at various developmental stages. (a) Time course of mortality of various instar larvae fed on leaves of different *RbPCD1pro::cryIAc* transgenic *Arabidopsis* lines as a percent of the total. Mortality values were analysed by one‐way ANOVA and compared using Duncan's Multiple Range Test (DMRT). Values on the bar carrying different letters are significantly different (α = 0.05). (b) Resistance of transgenic *RbPCD1pro::cryIAc Arabidopsis* plants against *H. armigera* larvae in various developmental stages as studied by leaf damage in detached (first and second instar larvae) and whole plant (third and fourth instar larvae) insect assay in three independent transgenic lines.

For detached leaf bioassays, plants of 10 transgenic lines (of 14) were randomly selected for neonate first instar larvae studies. All the 10 *RbPCD1pro::cryIAc* lines showed greater than 60% larval mortality after 24 h of bioassay and >80% mortality within 48 h of feeding the leaves (Figure 4a). In two transgenic AtPCDCry lines, 4‐2‐1 and 6‐1‐2, 90%–100% larval mortality was observed within 24 h of exposure while seven plants killed all larvae within 48 h. The results showed that the transgenic lines, despite having low basal levels of CryIAc (3%–6% of *CaMV35Spro* lines), induced it to sufficiently high enough levels upon wounding to kill all *Helicoverpa* larvae and confer complete protection. No significant mortality was observed in larvae fed on nontransgenic leaves which were severely damaged by larval feeding (Figure 4b).

Three transgenic lines (3, 4 and 6) that conferred 90%–100% mortality of neonate larvae within 24 h were selected for further bioassays with more advanced larval stages. As shown in Figure 4a, mortality of 2nd instar larvae on detached leaves of these lines was between 56%–80% within 48 h and reached 100% within 72 h of feeding. When studied with the 3rd instar larvae, these became inactive within 48 h of feeding on transgenic leaves and died between 72 h and 96 h. Whole plant bioassay from these selected transgenic lines showed 90%–100% mortality of *H. armigera* larvae after 96 h (Figure 4a). The amount of transgenic leaves consumed by larvae was very small compared to control plant leaves but sufficient to kill the 3rd instar larvae.

Unlike other larval stages, mortality of 4th instar larvae on transgenic leaves was much reduced by 5 days but was associated with weight reduction. Compared to control leaves, where a 304% increase in larval weight was observed in 5 days, a significant reduction in larval weight ranging from 7.6% to 44% of the initial larval weight was observed (Table 1). Even after 5 days, the transgenic plants showed little damage since the larvae, despite their size, refrained from feeding any further. They remained motionless for most part and died after 6–7 days. The results indicated that wound‐inducible expression of CryIAc in transgenic *Arabidopsis* was sufficient to confer protection even against larger larvae.

**Table 1:** Percent weight reduction of 4th instar larvae fed on *Arabidopsis*

Line	Larval weight (Mean ± SE)	Percent weight gained/lost (against initial larval weight) after 5 days feeding
Control	204.53 ± 4.07^a^	304%
AtPCDCry 3‐3‐1	62.23 ± 1.69^b^	−7.6%
AtPCDCry 4‐2‐1	48.91 ± 2.19^c^	−27.4%
AtPCDCry 6‐1‐2	37.66 ± 1.89^d^	−44%

Each value shows the mean weight of 25 larvae after 5 days of feeding. Average larval weight and percent weight reduction were analysed by one‐way ANOVA and compared using Duncan's Multiple Range Test (DMRT). Values in the column carrying different letters are significantly different. Initial average larval weight was 67.27 ± 1.25 mg.

Importantly, unlike previous reports, none of the transgenic lines expressing *RbPCD1pro::CryIAc* showed any abnormality in vegetative or reproductive growth (Figure S3) compared to control.

### Transgenic tomato expressing *cryIAc* under the wound‐inducible *RbPCD1pro* protect plants from insect larvae

The efficacy of *RbPCD1pro* to drive *cryIAc* expression was also tested in a commercially important Indian tomato, Pusa Early Dwarf. Three independent transgenic lines SlPCDCry 3, SlPCDCry 9, and SlPCDCry10, were selected for detailed studies. However, unlike in *Arabidopsis*, no plants expressing *cryIAc* under the *CaMV35S* promoter could be developed despite several efforts.

**Figure 5:**
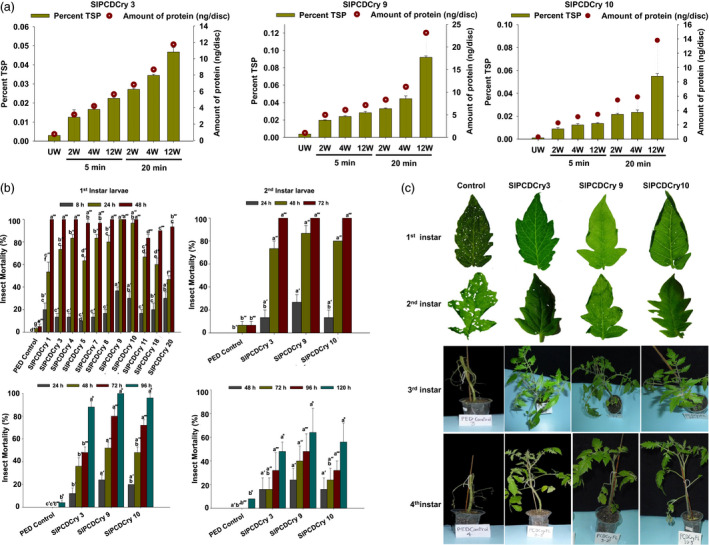
Wound‐inducible CryIAc estimation and efficacy of transgenic tomato plants expressing *cryIAc* under the *RbPCD1* promoter against *H. armigera* larvae at various developmental stages. (a) Wound‐inducible expression of CryIAc toxin under the control of *RbPCD1* promoter in different transgenic tomato lines. Expression was estimated in leaves of various transgenic lines as described in Figure 3a. Green bars represents %TSP on left *Y*‐axis and circles represent amounts in ng/disc (177 mm^2^ surface area) on right *Y*‐axis. UW‐unwounded, W‐ wounded. (b) Time course of mortality of various instar larvae fed on leaves of different *RbPCD1pro::cryIAc* transgenic tomato lines as a percent of the total. Mortality values were analysed by one‐way ANOVA and compared using Duncan's Multiple Range Test (DMRT). Values on the bar carrying different letters are significantly different (α = 0.05). (c) Resistance of transgenic *RbPCD1pro::cryIAc* tomato plants against *H. armigera* larvae in various developmental stages as studied by leaf damage in detached (neonate first and second instar larvae) and whole plant (third and fourth instar larvae) insect assay in three independent transgenic lines.

An estimation of the background and wound‐induced CryIAc protein produced in transgenic tomato leaves was performed using DAS‐ELISA as in case of *Arabidopsis*. As shown (Figure 5a), the transgenic tomato leaves had CryIAc background (unwounded) levels of 0.0042 ng/mm^2^ (SlPCDCry 3), 0.0054 ng/mm^2^ (SlPCDCry 9) and 0.0016 ng/mm^2^ (SlPCDCry10). Wounding caused a rapid increase in the CryIAc protein level with expression ranging from 0.981 to 1.995 ng/mm^2^ after 5 min of wounding in leaves. This was in the range observed for *Arabidopsis* and represented an increase in 284–613 fold over the unwounded background. The expression increased further to 2.57–3.66 ng/mm^2^ by 20 min, an increase in 680–1610 fold over the unwounded background. No CryIAc protein was detected in nontransgenic plants used as a negative control.

Insect bioassays were also performed to ascertain the toxicity of transgenic *RbPCD1pro::cryIAc* tomato plants towards *H. armigera* larvae at different stages using untransformed plants as control. Eleven transgenic plants (out of 22) from the T_0_ generation were randomly selected for insect bioassay on detached leaves using first instar neonate larvae. After 24 h of feeding, larval mortality in majority of transgenic lines was between 46%–96%, and reached 100% within 48 h of feeding (Figure 5b). The transgenic lines, SlPCDCry 9 and SlPCDCry 10 showed 97%–100% mortality within 24 h of exposure while six others displayed 100% insect mortality within 48 h of feeding. No mortality was observed in larvae feeding on nontransgenic leaves which showed far greater damage compared to transgenic leaves where hardly any damage was seen (Figure 5c).

The transgenic lines 3, 9, and 10, that conferred 100% mortality on neonate larvae within 48 h were selected for further insect bioassay with more advanced larval stages in the T_1_ generation. The selected transgenic lines displayed larval mortality of 70%–85% on 2nd instar larvae within 48 h and complete mortality within 72 h. These larvae caused severe damage on leaves of untransformed control plants but little or no damage on transgenic plant leaves (Figure 5b,c). Third instar larvae survived longer with larval mortality of 48%–80% after 72 h of feeding on transgenic plants and complete mortality only after 96 h (Figure 5b,c). The percentage of larval mortality of 4th instar larvae on transgenic plants after 5 days was lower and ranged between 28% and 32% (Figure 5b). Instead, a greater effect on weight reduction was observed in these larvae (Table 2). Compared to larvae fed on control leaves, where a 317% increase in larval weight was observed in 5 days, a significant reduction in larval weight, ranging from 3% to 35%, was observed compared to the initial larval weight (Table 2). As in case of *Arabidopsis*, the 4th instar larvae remained motionless for most part of the feeding trial and died after 6–7 days. The amount of transgenic leaves consumed by larvae even during their survival was very low unlike in controls where almost the complete plant was eaten by 4th instar larvae by the 3rd day.

**Table 2:** Percent weight reduction of 4th instar larvae fed on transgenic tomato

Line	Larval weight (Mean ± SE)	Percent weight gained/lost (against initial larval weight) after 5 days feeding
Control	219.63 ± 4.71^a^	317%
SlPCDCry 3	66.99 ± 1.34^b^	−3%
Sl PCDCry 9	44.67 ± 1.32^d^	−35%
SlPCDCry 10	58.61 ± 1.28^b^	−15%

Each value shows the mean weight of 25 larvae after 5 days of feeding. Average larval weight and percent weight reduction were analysed by one‐way ANOVA and compared using Duncan's Multiple Range Test (DMRT). Values in the column carrying different letters are significantly different. Initial average weight of larvae was 68.97 ± 0.88 mg.

The insect bioassay showed that 1st and 2nd instar larvae were more severely affected than older instars (3rd and 4th) as far as mortality was concerned although most of the 3rd and 4th instar larvae failed to damage the leaves. The results indicated that wound‐inducible expression of CryIAc in transgenic tomato conferred complete protection against the insects.

**Figure 6:**
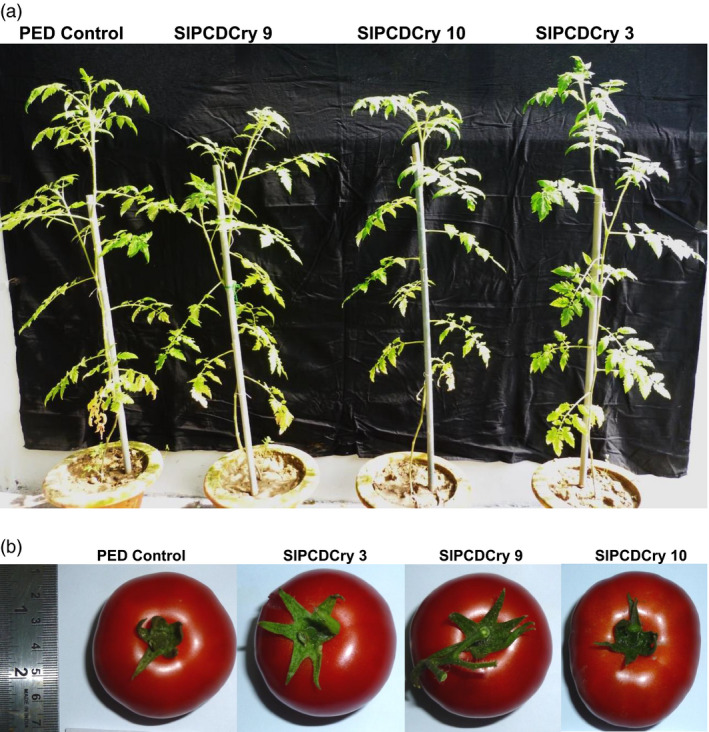
Phenotypes of transgenic tomato plants expressing *cryIAc* under the wound inducible *RbPCD1* promoter. (a) Whole plant growth after 3 months. (b) Fruit shape and size.

Importantly, no abnormalities were observed in transgenic plants during the entire course of plant growth. General plant growth and height, flowering time, fruit size and ripening pattern were not affected (Figure 6a,b).

Collectively, these results showed improved efficacy of CryIAc when expressed under the wound‐inducible *RbPCD1* promoter, heightened protection even against larger fourth instar larvae, reduced background levels compared to the *CaMV35S* promoter and no toxicity to plant growth and development unlike previous reports.

## Discussion

The efficacy of any transgene is governed largely by the promoter driving its expression. While constitutive promoters like *CaMV35S*, ubiquitin, actin, *rbcS* have been successful for driving high level expression of transgenes for abiotic and biotic stress tolerance, these are often associated deleterious effects or a yield drag in the absence of the stress (Bano‐Maqbool *et al*., 1998; Breitler *et al*., 2004; Gurr and Rushton, 2005; Kim *et al*., 2008; Koul *et al*., 2014; Rawat *et al*., 2011; Xia *et al*., 2010). Wound‐inducible promoters can effectively overcome these problems, provided these are strong and early acting. However, previous reports showed that the wound‐inducible *AoPR1* promoter of *Asparagus officinalis* (Warner *et al*., 1993), when used to express *cryIAc* in tobacco plants, accumulated the insecticidal protein only between 6 h and 72 h postwounding (Gulbitti‐Onarici *et al*., 2009). Similarly, the bell paper *fib* gene promoter, the *Stylosanthes Shpx6b* peroxidase promoter, and the *Populus win3.12* promoter showed wound‐inducible expression only 20–24 h after wounding (Chen *et al*., 1998; Hollick and Gordon, 1995; Perera and Jones, 2004; Yevtushenko *et al*., 2004). The wound‐inducible maize protease inhibitor *mpiC1* promoter when used to express a synthetic *cry1Ac* toxin in sorghum showed low accumulation of toxic protein only 12 h postwounding (Girijashankar *et al*., 2005). The rice *OsDof1* promoter in contrast, showed wound‐inducible *GUS* expression within minutes of wounding in transgenic *Arabidopsis* and rice (Park *et al*., 2014). However, since the GUS histochemical reaction did not include cycloheximide, one cannot rule out GUS expression during the overnight incubation for color development in these studies. Many of these promoters are specific to a plant and cannot be used in others e.g. the *WIP1* promoter from maize conferred wound response in rice but not in tobacco (Zhang *et al*., 2013). Often dicot promoters show reduced efficiency in monocots and *vice versa* (Christensen *et al*., 1992; Wilmink *et al*., 1995) restricting their use for transgene expression. An alternate strategy for insect resistance is high level expression of Cry toxins in plastids which also has an added advantage of transgene containment due to the maternal inheritance of the plastid (De Cosa *et al*., 2001; Kota *et al*., 1999). However, chloroplast transformation is restricted to a few plants due to a high level of standardization required.

In comparison, the *RbPCD1* promoter confers a strong early acting wound‐inducible expression in several different plants. Detailed analysis in *Arabidopsis* and tomato showed induction, *GUS* transcript accumulation and *GUS* activity within 5–20 min of mechanical wounding in the transgenic *Arabidopsis* leaves (Figure 1). A quantitative measure of *GUS* transcript level using qRT‐PCR showed an induction of 50–150 fold after 5 min and 150–550 fold after 20 min of wounding. This transcriptional increase was also associated with high levels of GUS protein as reflected from the histochemical results indicating that both transcription and translation were responsible for the rapid GUS increase under the *RbPCD1* promoter. The use of cycloheximide during color development ensured that all activity estimated during color development represented GUS protein produced only within the 5–20 min period of wounding. The activation of the promoter at the transcriptional level as well as translational level within 5 min of wounding makes it one of the fastest promoters to be studied so far.

Wound induction was also seen in transgenic chickpea leaves as well as agro‐infiltrated rose petals, Gladiolus tepals, cotton sepals, and tobacco leaves (Figure S1). This indicates that wound‐inducible *cis* elements within this region are recognized by the wound machinery across a wide range of plants that include both monocots and dicots and across a wide range of tissues such as leaves, sepals, and petals. This is important since it potentially extends the applicability of the promoter across monocots and dicots and in commercially important families such as Brassicaceae, Solanaceae, Leguminaceae, Rosaceae. The promoter is also activated in response to attack by sucking pests such as aphids and by chewing insects like *Helicoverpa* specifically at the points of insect damage (Figure 2A) indicating its potential to target both sucking and chewing type insects using suitable insecticidal toxins against each. Its activation by JA might explain its activation in a wide range of plants where JA functions as a primary hormone for wound induction and herbivory (Erb *et al*., 2012). The *RbPCD1* promoter was shown to be rapidly induced by mechanical wounding in all aerial parts of *Arabidopsis*. At no stage of development was expression seen in the absence of wounding except in some inflorescence parts. This suggests that the *RbPCD1* promoter could be used in a developmental stage‐independent manner unlike the *CaMV35S* promoter which, despite its constitutive nature, has been reported to show reduced expression at the time of flowering in cotton plants leading to susceptibility to insect attack at that stage (Kranthi *et al*., 2005).

Constitutive promoters drive gene expression independently of external stimuli leading to accumulation of high levels of toxic protein at all stages of development. This has been shown to be responsible for abnormalities in development in transgenic cotton (Rawat *et al*., 2011) and tomato plants (Koul *et al*., 2014, 2015) expressing the Bt toxins and their variants. The possible lethality to plants expressing high levels of the toxin under the *CaMV35S* promoter invariably leads to selection of plants expressing the Bt toxin at lesser amounts than is desirable for effective protection against insect attack (Rawat *et al*., 2011). In the present study, no transgenic plants expressing *cryIAc* under the *CaMV35S* promoter could be raised in spite of screening four times as many plants as for the *RbPCD1* promoter. Strong constitutive expression of a toxic protein, even in the absence of its need, creates a high metabolic load on plants. This can lead to diversion of essential resources towards toxin production and affect other processes – developmental as well as adaptive. For instance, one of the first responses upon abiotic and biotic stress signalling is to reduce normal growth processes and divert resources towards fighting the stress. The opposite actions of stress hormones like JA/SA/ethylene/ABA versus growth hormones like auxin/GA often leads to either suppression of growth pathways during stress or of stress pathways during growth (Groszmann *et al*., 2015; Machado *et al*., 2013; Pandey *et al*., 2017). In such conditions, the presence of a strong constitutive promoter like *CaMV35S* that continuously synthesizes an insecticidal toxin protein regardless of a plants’ needs, may compromise defense or abiotic stress responses by reducing the resources available for fighting these, thereby increasing susceptibility to the stresses. In the absence of the stress, it may lead to a yield drag (Bano‐Maqbool *et al*., 1998; Breitler *et al*., 2004; Kim *et al*., 2008; Xia *et al*., 2010). In contrast, a wound‐inducible promoter would remain silent during normal developmental processes and during responses to any stress (other than that which causes wounding) and would thus not interfere with these responses. This is borne out from the present studies through expression of *RbPCD1pro::cryIAc* in two different transgenic plants, *Arabidopsis* and tomato, where expression of the CryIAc toxin was dependent on wounding. The base level of CryIAc protein in transgenic plants was less than a fifth of the best *CaMV35S* plants while induced levels of the CryIAc protein were 5–80 times higher than the *CaMV35S* lines per unit area and more than 300–1600 times higher than the unwounded background depending on the time. The time period of activation was early enough to prevent insect damage on leaves beyond the first few nibbles by small larvae and strong enough to produce the toxic Bt protein at levels that caused 100% mortality even up to third instar larvae within that period. Interestingly, even the normally voracious fourth instar larvae failed to eat the leaves and remained motionless. This indicates that the amount of protein produced upon wounding was toxic even to the much larger fourth instar larvae. Importantly, the expression of toxic gene under this promoter allowed plant growth without any abnormality at any stage of development in *Arabidopsis* as well as tomato unlike previous reports.

In conclusion, these studies demonstrate the efficacy of *RbPCD1pro* as one of the earliest acting wound‐inducible promoters in plants with widespread wound‐inducible expression in different aerial tissues and potential applicability across several dicot and monocot families compared to commonly used constitutive promoters.

## Experimental procedures

The wound‐inducible *RbPCD1* promoter was isolated from *Rosa bourboniana* during an abscission‐related study. *Arabidopsis thaliana,* ecotype Columbia (Col‐0) and tomato (*Solanum lycopersicon* var. Pusa Early Dwarf) were used for validation of the efficacy of wound‐inducible promoter. All the plants were grown in culture room/glasshouse at ~23°C with a 16/8 h light/dark period, at ~78% relative humidity.

### Isolation of *RbPCD1* promoter

The *RbPCD1* promoter (sequence provided in Patent No 3866/DEL/2014 filed and WO2016103279A1) was isolated from a rose genome walking library as a 523 nt region upstream of the *RbPCD1* translation initiation codon, identified during a petal abscission study (Tripathi, 2006). It was cloned in pBI101 and used for GUS expression studies (Figure S2A) in *Arabidopsis* and other plants.

Real‐time PCR to study wound‐induced *GUS* expression was carried out with primers GUSF‐SQ (AAAGGTTGGGCAGGCCAGCG) and GUSR‐SQ (GGCGTATAGCCGCCCTGATG C) on an ABI Prism 7000 machine (Applied Biosystems, Foster City, CA) using Power‐up SYBR Green mastermix in three biological and technical replicates. The analysed data were the mean of biological and technical triplicates. Relative gene expression was calculated using the $2^{-\Delta \Delta C_{T}}$ method (Livak and Schmittgen, 2001) and normalized against *AtUBIQUITIN10* amplified with the primers AtUbiq10‐F (GGCCTTGTATAATCCCTGATGAATAAG) and AtUbiq10‐R (AAAGAGATAACAGGAACGGAAACATAG).

### Wounding and agro‐infiltration of plants

Transgenic plant leaves were wounded by puncturing with forceps (wound area 1 mm^2^) and kept on the plant for 5 and 20 min followed by color development as described (Gattolin *et al*., 2006). Cycloheximide (1.8 mm) was included in the color development reaction as a protein translation inhibitor to ensure that the GUS protein was synthesized only during the 5–20 min time course of wounding and not later during the development of color. For JA (100 μm) and SA (2 mm) treatments, excised leaves of 30‐day‐old transgenic plants were incubated with the hormones or mock (water or 0.1% ethanol) for 3 h before color development.

Agro‐inoculation of plants such as rose, cotton, tobacco, and gladiolus was carried out as described by Tripathi *et al*. (2009). Thereafter, tissues were kept on the plant for 2 days and analysed for GUS expression after wounding. Agrobacterial suspension containing pBI101 (no promoter) and pBI121 (GUS driven by CaMV35S promoter) were injected as above and used as negative and positive controls, respectively.

### Generation of the *RbPCD1pro::cry1Ac* fusion construct

To study the ability of the *RbPCD1* promoter to drive *cry1Ac* expression in a wound‐specific manner, the 523 nt region of *RbPCD1pro* was amplified using the primer PCDpro‐WlcryF (GGATCCTAACCGCTAGGCAGTGAGC) in combination with PCDpro‐WIcryR (AAGCT TCTTCTTCTCTGTTACCTGAAA). Amplified fragments were cloned in pTZ57R/T, sequence confirmed and the fragment excised from the vector using HindIII. The excised *RbPCD1pro* was used to replace the *CaMV35S* promoter from its source vector in the expression cassette *35Spro::cry1Ac* (Koul *et al*., 2015) to obtain *RbPCD1pro::cry1Ac* (Figure S2B). The vectors carrying *cryIAc* under the wound‐inducible *RbPCD1* and the *CaMV35S* promoters were introduced into Agrobacterium GV3101 by the freeze‐thaw method (Hofgen and Willmitzer, 1988). *Arabidopsis* was transformed with these constructs by the floral dip method (Clough and Bent, 1998) and tomato via *Agrobacterium‐*mediated transformation (McCormick *et al*., 1986). Transformants, selected on 50 μg/mL kanamycin, were confirmed by PCR. Progeny of at least three independent lines each of transgenic *Arabidopsis*, (AtPCDCry 3‐3‐1, 4‐2‐1, and 6‐1‐2, T_3_ generation) and tomato, (SlPCDCry 3, SlPCDCry 9, and SlPCDCry10, T_1_ generation) were studied in detail.

### Southern analysis of transgenic plants

DNA from transgenic *Arabidopsis* and tomato plants expressing *cryIAc* under the *CaMV35S* and *RbPCD1* promoters was isolated as described (Asha *et al*., 2007). About 25–30 μg DNA was restriction‐digested with HindIII, electrophoresed on a 0.8% TAE‐agarose gel and transferred to a Hybond‐N nylon membrane by vacuum transfer on a Vacugene blot (Pharmacia) as described (Sambrook *et al*., 1989). A 3.3 kb region of the *cryIAc* gene encompassing the regions between 1–1305, 1305–2509, and 2381–3342 nt in three fragments was radio‐labelled by PCR with ^32^PdCTP and used as a probe. Hybridization was performed as described (Sane *et al*., 1994) and the membrane exposed to X‐ray film for 3–7 days as per intensity of signal.

### Insect bioassay

The efficacy of transgenic *Arabidopsis* and tomato plants expressing *RbPCD1pro::cry1Ac* was checked against different larval stages of *Helicoverpa armigera* by no choice insect bioassays using detached leaves and whole plants of 45‐day‐old transgenic *Arabidopsis* (T_1_ and T_3_ generation) and 2‐month‐old tomato (T_0_ and T_1_ generation) plants. Untransformed plants were used as controls. Leaves of independent transgenic lines were placed in boxes with a moist blotting paper and 10 larvae each of first or second instar placed on these. Mouths of boxes were covered with moist muslin cloth to maintain humidity and kept under 16/8 h light/dark regime at 25°C. Each experiment (repeated thrice) contained five replicates of leaves of independently grown transgenic lines.

Whole plant insect assay was performed independently with third and fourth instar larvae (five plants/line). Five larvae were released on the leaves of control and transgenic plants and enclosed within a plastic bag. Insects were allowed to feed on plants for four (*Arabidopsis*) or 5 days (tomato) by which time control plants were damaged considerably. Percent mortality, calculated as the number of dead insects/total number of insects ×100, was evaluated thereafter. Surviving larvae were collected and differences in weight compared to those fed on un‐transformed *Arabidopsis*/tomato plants recorded. All experiments were repeated thrice.

For studies with the sucking pest, transgenic *Arabidopsis* plants expressing *RbPCD1pro‐GUS* were exposed to insects of *Myzus persicae,* collected from a culture room with *Arabidopsis* plants showing chance infection with these insects. About 8–10 insects were allowed to feed on leaves of the transgenic plants. After 2 days, leaves of infested plants were tested for GUS expression.

### Quantitative estimation of recombinant Cry1Ac protein in plants

Intact leaves of transgenic plants were wounded rapidly with a pair of forceps (wound area of 1 mm^2^) and leaves left on plant for 5/20 min after wounding. Thereafter leaf discs encompassing the wounded region were cut using a chopper (1.5 cm diameter) and immediately frozen in liquid N_2_ along with control (unwounded) leaf discs separately. Protein was isolated from the discs, homogenized in ice‐cold protein extraction buffer (100 mm Tris‐HCl, pH 8.0, 500 mm NaCl, 5 mm DTT, 2 mm PMSF, and 5 mm EDTA) and centrifuged at 12 000 ***g*** (10 min, 4°C). Total soluble protein (TSP) was determined as per Lowry *et al*. (1951). Quantitative estimation of Cry1Ac protein was performed using double antibody sandwich enzyme‐linked immunosorbent assay (DAS‐ELISA) on an antibody‐coated 96‐well microtiter plate (Peroxidase label, Agdia, Elkhart, IN) with the kit‐provided Cry1Ac from *Bacillus thuringinesis azawaii* as positive control and cell free extract of nontransformed plant as negative control. For quantification, a standard curve of varying amounts of quantified CryIAc protein (expressed from the source cDNA and obtained from Dr PK Singh, CSIR‐NBRI) was prepared. Quantification of the plant‐expressed protein from *RbPCD1pro::cryIAc* or *CaMV35Spro::cryIAc* plants was carried out against this reference. The Cry1Ac protein produced per wound (1 mm^2^) under *RbPCD1pro* was estimated by subtracting the background of CryIAc under unwounded conditions in the leaf disc. This was compared with protein produced under the constitutive *CaMV35Spro* within the disc (177 mm^2^ area) calculated as below:

Amount of CryIAc in unwounded transgenic leaf disc (177 mm^2^) = *x* ng
Background level of CryIAc expressed in *RbPCD1pro::cryIAc* plant/mm^2^ = *x*/177
Amount of CryIAc in transgenic leaf disc with 2 wounds (1 mm^2^ each) = *y* ng
Amount of CryIAc per wound (per mm^2^) = (*y*−*x*)/2
Amount of CryIAc in a *CaMV35Spro::cryIAc* leaf disc = *z* ng
Amount of CryIAc expressed in *CaMV35Spro::cryIAc* plant/mm^2^ = z/177

### Statistical analysis

The data were statistically analysed by applying One way‐ ANOVA (*P* < 0.05) and means were compared using Duncan's Multiple Range Test (DMRT) by using SPSS software.

## Conflict of interest

The authors declare no conflict of interest.

## Supporting information


**Figure S1** Wound‐induced activation of *RbPCD1pro*::*GUS* in different plants.
**Figure S2a** Schematic representation of the *RbPCD1pro::GUS* expression cassette in pBI101 backbone used for plant transformation.
**Figure S2b** Schematic representation of the *RbPCD1pro::cry1Ac* expression cassette in pBI101 backbone used for plant transformation.
**Figure S3** Growth phenotypes of transgenic *Arabidopsis* plants expressing *cryIAc* under the *RbPCD1* promoter.
