# Supplementary material for: A strong early acting wound‐inducible promoter, RbPCD1pro, activates cryIAc expression within minutes of wounding to impart efficient protection against insects
Source: Plant Biotechnol J. 2019 Feb 26;17(7):1458–70. doi: 10.1111/pbi.13071 (PMC6576099; doi:10.1111/pbi.13071)
Supplement: Supplementary file 1 — Figure S1 Wound‐induced activation of RbPCD1pro::GUS in different plants. Figure S2a Schematic representation of the RbPCD1pro::GUS expression cassette in pBI101 backbone used for plant transformation. Figure S2b Schematic representation of the RbPCD1pro::cry1Ac expression cassette in pBI101 backbone used for plant transformation. Figure S3 Growth phenotypes of transgenic Arabidopsis plants expressing cryIAc under the RbPCD1 promoter. [file PBI-17-1458-s001.pdf]

A strong early-acting wound-inducible promoter, *RbPCD1pro*, activates *cryIAc* expression within minutes of wounding to impart efficient protection against insects

Saurabh Prakash Pandey[1,2], Amar Pal Singh[1,3], Shruti Srivastava[1], K. Chandrashekar[4,5] , Aniruddha P. Sane[1,2*]

[1]Plant Gene Expression Lab, and [4]Genomics and Molecular Biology Division, CSIR-National Botanical Research Institute, Lucknow-226001 India

[2]Academy of Scientific and Innovative Research (AcSIR), Ghaziabad-201002, India

[3]Current address: National Institute for Plant Genome Research, New Delhi, India

[5]Current address: IARI Regional Centre, Aundh, Pune - 411067, India

Supplementary figures

Supplementary Figure S1. Wound-induced activation of *RbPCD1pro*::*GUS* in different plants. Histochemical analysis of GUS activity in *RbPCD1pro*::*GUS* expressing transgenic chickpea and agro-injected cotton sepal, tobacco leaf, *Gladiolus* tepal and rose petal after wounding.

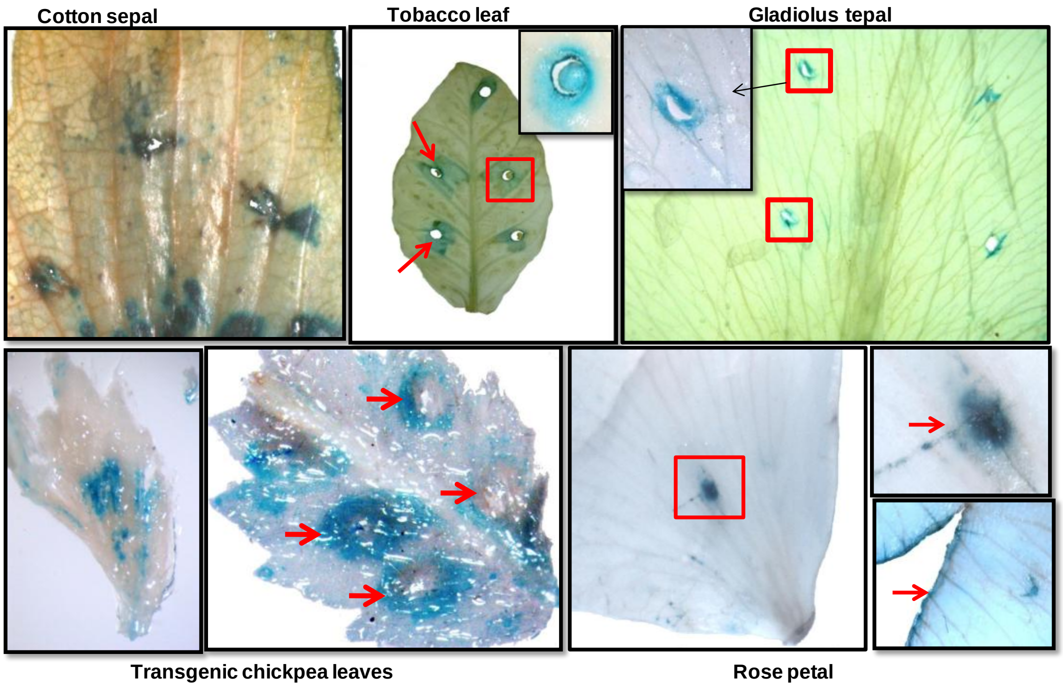

Supplementary Figure S1

Supplementary Figure S2A. Schematic representation of the *RbPCD1pro::GUS* expression cassette in pBI101 backbone used for plant transformation.

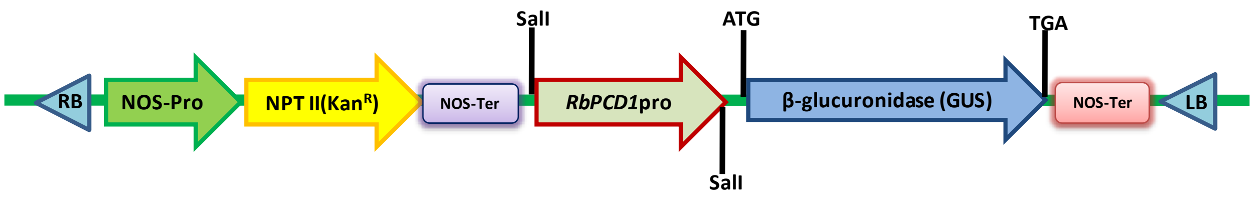

Supplementary Figure S2A

Supplementary Figure S2B. Schematic representation of the *RbPCD1pro::cryIAc* expression cassette in pBI101 backbone used for plant transformation.

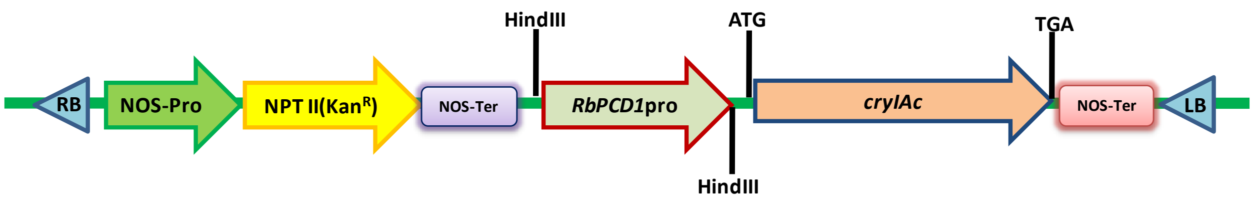

Supplementary Figure S2B

Supplementary Figure S3. Growth phenotypes of transgenic Arabidopsis plants expressing *cryIAc* under the *RbPCD1* promoter.

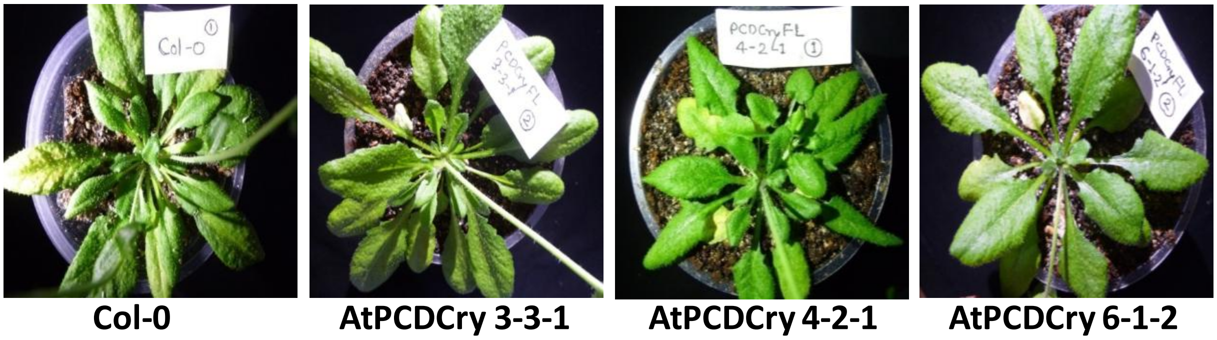

Supplementary Figure S3
